# An optimized test bolus for computed tomography pulmonary angiography and its application at 80 kV with 10 ml contrast agent

**DOI:** 10.1038/s41598-020-67145-9

**Published:** 2020-06-23

**Authors:** Huiming Wu, Xiao Chen, Hao Zhou, Bin Qin, Jian Cao, Zhaochun Pan, Zhongqiu Wang

**Affiliations:** Department of Radiology, the Affiliated Hospital of Nanjing University of Chinese Medicine, Nanjing, China

**Keywords:** Vascular diseases, Embolism

## Abstract

Computed tomography pulmonary angiography (CTPA) is usually used for pulmonary embolism (PE) detection. However, the determination of scan timing remains a challenge due to the short scan duration of CTPA. We aimed to develop an optimized test bolus to determine scan delay in CTPA. The time-enhancement curves were obtained by measuring the enhancement within a region of interest in the main pulmonary artery and vein. A total of 70 patients were randomly divided into two groups (n = 35 each): the control group underwent CTPA using the test bolus approach and the test group underwent CTPA using the biphasic time-enhancement curves approach. Tube voltages of 100 kVp and 80 kVp and 20 ml and 10 ml contrast agent were adopted in the control and test groups, respectively. The CT numbers, image quality, PE detection was evaluated. There was a point of intersection between the pulmonary artery and vein test bolus enhancement curves. The scan delay time (T_DELAY_) was obtained based on the time at intersection (T_CROSS_) and the scan duration (T_SD_): T_DELAY_ = T_CROSS_ − T_SD_. The mean CT numbers for pulmonary vein in the control were higher than those in the test group (all p < 0.001). The image quality for the pulmonary arteries in the test group was better than that in the control group (p < 0.01), with artifact reduction in the superior vena cava. Segmental PE could be detected using the optimized protocol. The radiation dose and iodine load in the test group were all lower than those in the control (p < 0.01). We established an approach to calculate the scan delay of CTPA, and this approach could be used for CTPA at 80 kVp with 10 ml contrast agent.

## Introduction

Computed tomography pulmonary angiography (CTPA) is a critical imaging approach for pulmonary embolism (PE) detection with high specificity and sensitivity^1,2^. Most of the previous studies used the bolus tracking system (hounsfield unit of the region of interest positioned within the main pulmonary artery) or a test bolus to start the scan^3,4^. The scan protocols are complex and challenging due to the application of multidetector CT^3^. The optical scanning time is difficult to predict because the hemodynamic profile of patients are unknown^4^. The contrast material arrival time was regarded as the diagnostic scan delay in a single-detector CT angiographic scan^5^. In addition, the peak arterial enhancement can be missed when the scan delay is excessively extended^4^. For fast multidetector CT, an additional delay is usually used^6^. Low kilovoltage scanning based on a small volume of contrast media also affects the determination of the scanning time^7^. Moreover, venous contamination usually occurred in the bolus tracking system^8^. Subclavian vein (SV) and superior vena cava (SVC) artifacts influence on the PE detection. However, the methods of determining the delay varies for different investigators.

Efforts have been made to reduce the radiation dose, such as using automated tube current modulation dose systems and reductions in the voltage from 120 kVp to 100 or 80 kVp^9–11^. Moreover several studies have also investigated the image quality of CTPA using both low-voltage scanning and low doses of contrast medium^11^. The risk of contrast-induced nephropathy (CIN) is low^12^. However, one should practice caution for patients with high risk, such as those with severe chronic kidney disease, because contrast-induced nephropathy (CIN) may occur^13–16^. The European Society of Urogenital Radiology guidelines recommend the avoidance of unnecessarily high doses of contrast media^17^. Recently, several studies reported that CT angiography with a reduced iodine load (20–30 ml) was also available in CTPA without compromising diagnostic image quality^11,18,19^. However, no studies using a reduced amount of contrast agent have been reported.

The test bolus method was based on the attenuation of the main pulmonary artery. To the best of our knowledge, the attenuation of the pulmonary vein during the scanning has not been considered. We hypothesized that it may supply information to determine the time of scan start and may be valuable for personalized CTPA, which is the current trend in individualized dianostic^18^. In the present study, we established a protocol to determine the scan delay time by using the biphasic time-enhancement curve in the pulmonary artery and vein. We tested it using a very low volume of contrast media (10 ml).

## Results

### The characteristics of patients

The characteristics of the patients are listed in Supplementary Table s1. There were no significant differences in gender, age, height, weight and BMI between the test group and the control group.

### The time-enhancement curves

**Figure 1:**
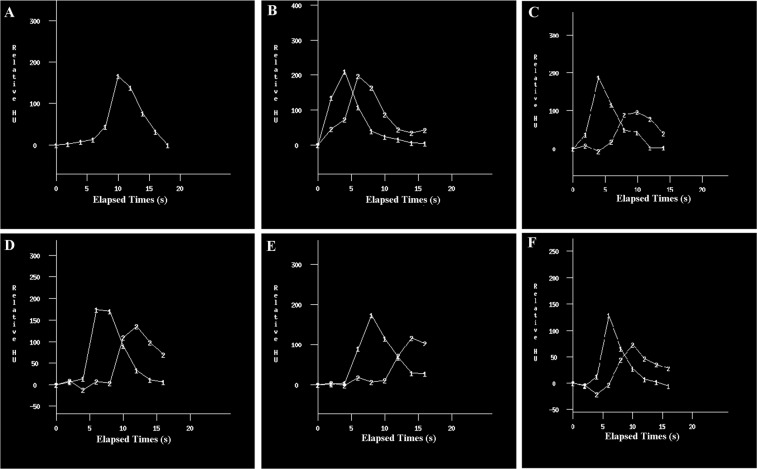
The time-enhancement curves in control (**A**) and test group (**B–F**). The time-enhancement curves were obtained by measuring the enhancement within a region of interest in pulmonary artery and vein. The biphasic time-enhancement curves were obtained in test group (**B–F**). There was an intersection between the two curves. In order to decrease the vein enhancement, the scan should be finished before the time at cross point. The scan delay (T_DELAY_) was calculated by using the following equation: Time at cross point (T_CROSS_)-scan duration (T_SD_). Line 1 was the time-enhancement curves of pulmonary artery; Line 2 was the time-enhancement curves of pulmonary artery of vein.

The time-enhancement curves of the pulmonary artery and vein are shown in Fig. 1. The curves varied in different patients. To finish all scans before the point of intersection (T_CROSS_), we established the following equation: scan delay time (T_DELAY_) = T_CROSS_− scan duration (T_SD_). We calculated the T_DELAY_ in six patients based on the time-enhancement curves (Fig. 1, Supplementary Table s2). The data showed that the T_DELAY_ varied in different patients (3–7 s). We also calculated the scan delay using the test bolus methods. There were differences in T_DELAY_ between the test bolus method and our protocol.

### Image quality evaluation

**Figure 2:**
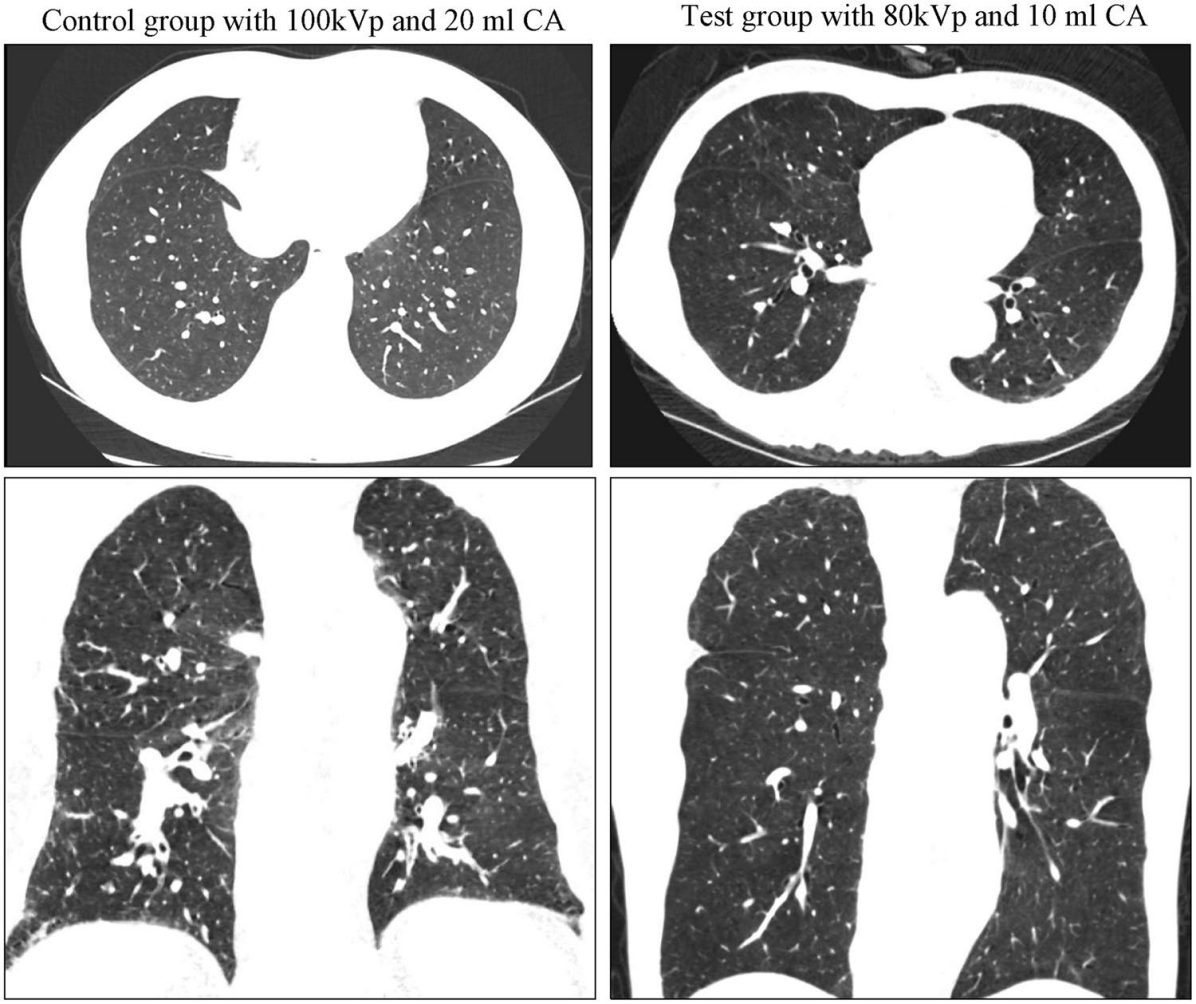
Lung window images in the control and test groups. Axial (upper) and coronal (lower) lung window CT image both clearly show the image quality was similar between the control and test groups. CA: contrast agent.

**Figure 3:**
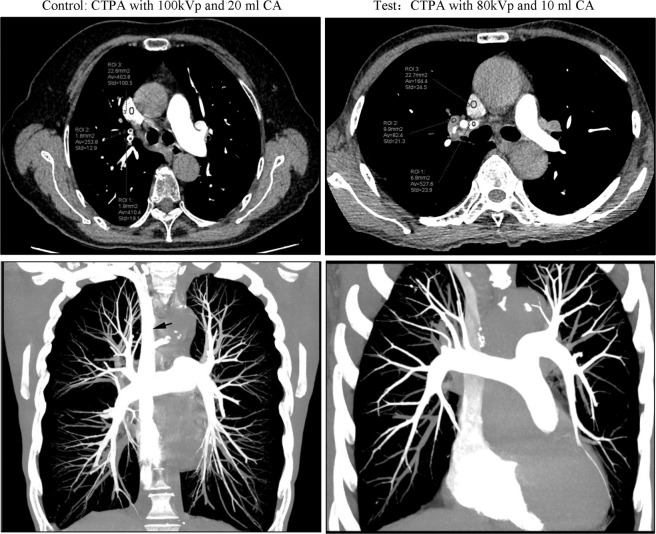
Axial (upper) and maximum intensity projection (lower) computed tomography pulmonary angiography (CTPA) images in the control (a 76-year-old man) and test groups (a 77-year-old man). Artifacts of superior vena cava (SVC) was observed in the control group (arrow) and the CT number of SVC was 403. The CT number of SVC (78.4HU) in test group (arrow head) was lower than that in the control group. In addition, the CT number of pulmonary vein in upper and lower lung lobes in control group were all higher than those in control group. CA: contrast agent.

**Figure 4:**
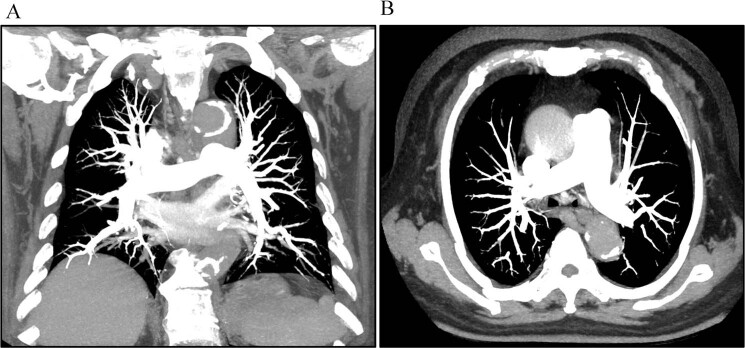
Computed tomography pulmonary angiography (CTPA) (**A**: coronal; **B**: axial) of a 77-year-old female in the control group using test bolus method. The scan start was delayed. The venous contamination was severe.

Table 1 illustrates the subjective evaluation of image quality of PA, image quality at the lung window and PV artifact in the two groups. No significant differences were observed in the image quantity at the lung window (Fig. 2). Both readers showed that the image quality of PA in the test group was better than that in the control group (p < 0.001). Moreover, fewer artifacts in SVC and SV were observed in the test group than in the control group (p < 0.001) (Table 1. Figs. 3, 4). Figure 4 shows a case of CTPA images with pulmonary venous contamination in the control group.

**Table 1:** Subjective evaluation of image quality and artifact.

		Score	Test group	Control group	χ2	P value
Pulmonary artery	Reader 1	5	28(80%)	10(29%)	22.32	<0.001
		4	7(20%)	13(37%)		
		3	0	12(34%)		
		2	0	0		
		1	0	0		
	Reader 2	5	32(91%)	15(43%)	19.55	<0.001
		4	3(9%)	12(34%)		
		3	0	8(23%)		
		2	0	0		
		1	0	0		
Lung window image	Reader 1	1	25	20	1.984	0.372
		2	10	15		
		3	0	0		
	Reader 2	1	21	21	0	1.0
		2	14	14		
		3	0	0		
Artifact of SV and SVC	Reader 1	SVC	6(17%)	28(80%)		<0.001
		SV	5(14%)	31(89%)		<0.001
	Reader 2	SVC	9(26%)	26(74%)		<0.001
		SV	4(11%)	28(80%)		<0.001

SV: subclavian vein; SVC: superior vena cava.

Subsequently, we also compared the CT values, signal noise ratio (SNR) and contrast noise ratio (CNR) of the main pulmonary artery (MPA), left and right pulmonary artery (LPA and RPA), left upper and lower lobe pulmonary arteries, the apical segment in the left upper lobe and the companion veins, and the basal segment in left lower lobe and the companion veins (Table 2). The CT values, SNR and CNR of the above arteries and veins in the test group were all lower than those in the control group (all p < 0.001). In addition, no significant differences were found in the CT values and SNR of the lung, image noise or CT values of the muscle between the two groups.

**Table 2:** Objective image measurement.

			Test group	Control group	P value
MPA		CT number	317.3 ± 53.9	395.9 ± 86.5	<0.001
		SNR	26.8 ± 7.6	38.1 ± 13.9	<0.001
		CNR	21.9 ± 7.1	32.9 ± 12.8	<0.001
LPA		CT number	316 ± 52.5	388.2 ± 88.6	<0.001
		SNR	26.7 ± 7.6	37.5 ± 13.9	<0.001
		CNR	21.8 ± 6.9	32.3 ± 12.9	<0.001
RPA		CT number	320.1 ± 53.2	381.5 ± 87.2	0.001
		SNR	27.1 ± 7.9	36.9 ± 15.9	0.001
		CNR	22.1 ± 7.3	31.8 ± 12.8	<0.001
Left upper lobe PA		CT number	320.3 ± 54.8	412.1 ± 100.2	<0.001
		SNR	27 ± 7.3	39.9 ± 15.9	<0.001
		CNR	22.1 ± 6.8	34.8 ± 14.8	<0.001
The apical segment	Artery	CT number	308.9 ± 53.8	411.3 ± 118.1	<0.001
		SNR	26 ± 7.2	39.9 ± 16.9	<0.001
		CNR	21.1 ± 6.7	34.8 ± 14.1	<0.001
	Vein	CT number	92.2 ± 44.2	233.8 ± 121.3	<0.001
		SNR	7.8 ± 4.1	21.9 ± 12.4	<0.001
		CNR	2.8 ± 4.1	16.8 ± 11.7	<0.001
Left lower lobe PA		CT number	335.5 ± 50.9	408.1 ± 113.7	0.001
		SNR	28.2 ± 7.4	39.7 ± 17.1	0.001
		CNR	23.3 ± 6.7	34.6 ± 16.1	<0.001
Basal segment	Artery	CT number	326.7 ± 51.2	393.2 ± 121.8	0.005
		SNR	27.5 ± 6.8	37.6 ± 14.7	0.001
		CNR	22.5 ± 6.1	32.4 ± 13.8	<0.001
	Vein	CT number	51.7 ± 25.7	125.3 ± 103.2	<0.001
		SNR	4.4 ± 2.6	11.6 ± 9.7	<0.001
		CNR	−0.5 ± 2.7	6.5 ± 9.5	<0.001
Paraspinal muscle		CT number	58.2 ± 11.7	53.2 ± 10.4	0.065
Noise		SD value	12.4 ± 2.5	11.3 ± 3.5	0.158
Left pulmonary lobes		CT number	−857.2 ± 49.4	−858.3 ± 40.3	0.547
		Noise	30.6 ± 9.9	30.1 ± 10.1	0.646
		SNR	−31.2 ± 10.9	−32.1 ± 11.7	0.828
Right pulmonary lobes		CT number	−832.8 ± 62.5	−822.3 ± 61.6	0.852
		Noise	33.6 ± 11.6	35.7 ± 10.2	0.224
		SNR	−27.6 ± 8.6	−25.3 ± 8.5	0.322

MPA: main pulmonary artery; LPA: left pulmonary artery; RPA: right pulmonary artery.

### Detection of PE

**Figure 5:**
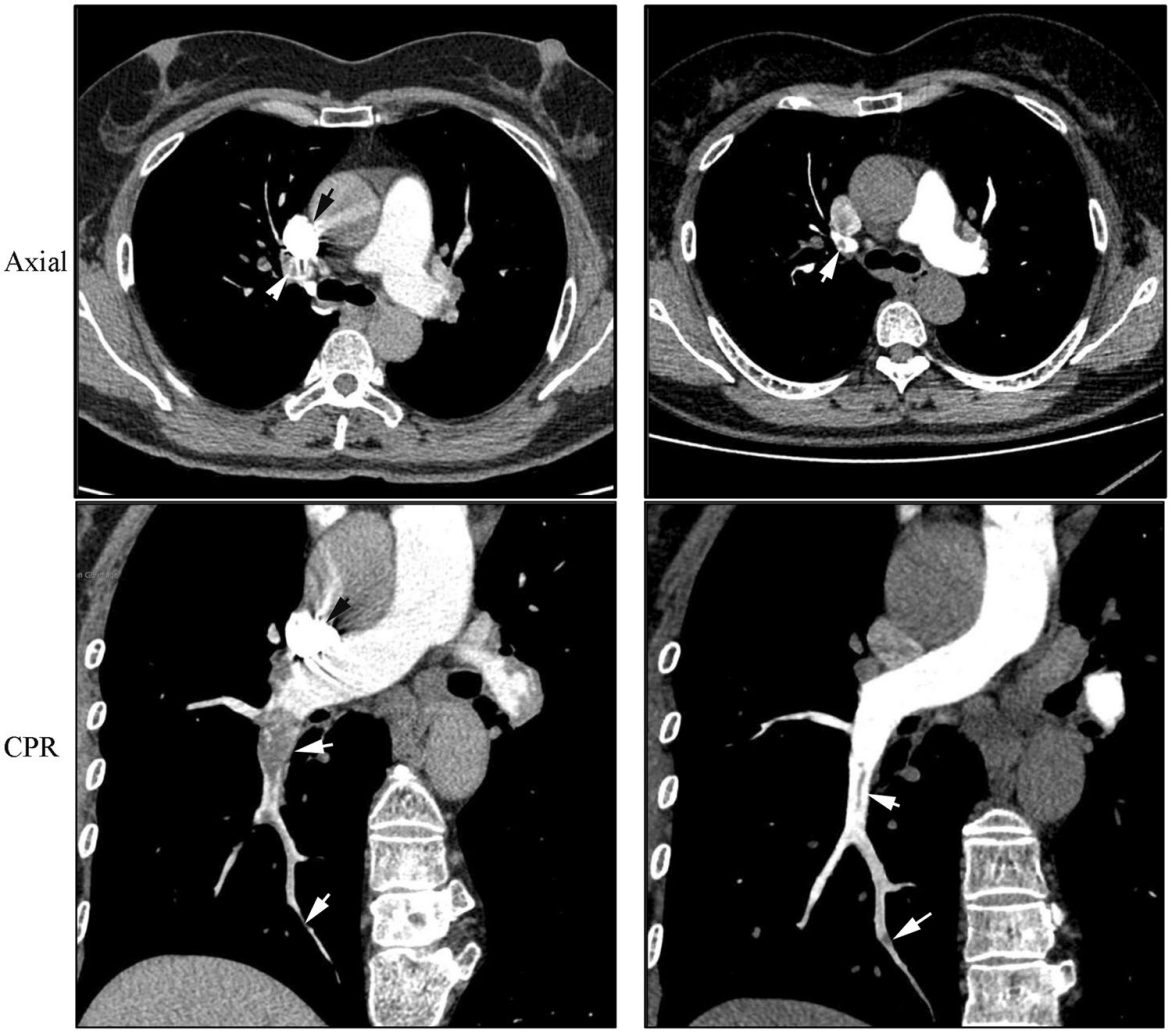
A 58-years old female patient who underwent computed tomography pulmonary angiography (CTPA) examinations both using test bolus method (Left) and the optimized protocol (right). She firstly underwent the CTPA using test bolus method. The artifacts of superior vena cava (SVC) were occurred (black arrow) in axial and curve plane reformation (CPR) images which influenced embolism detection (arrow head). During the follow-up at 6th month, the CTPA was performed using the optimized protocol (biphasic time-enhancement curve). No artifacts of SVC was observed. The two methods both detected the pulmonary embolism at lobar PA and segmental PA (white arrow).

PE was detected in 6 and 5 patients in the test and control groups, respectively. The number of PE is listed in Table 3. PE in the lobar PA and segmental PA both could be detected using the optimized protocol (test group) and the test bolus method (control group). We showed a patient who underwent CTPA using both the test bolus method and the optimized protocol (Fig. 5). The SVC artifacts occurred in the axial and curve plane reformation images which influenced embolism detection. The CTPA images indicated that they had similar detection performance.

**Table 3:** Pulmonary embolism detection.

	Test group		Control group	
	Reader 1	Reader 2	Reader 1	Reader 2
MPA	0	0	0	0
LPA and RPA	0	0	4	6
Lobar PA	11	10	11	12
Segmental PA	18	21	31	34
Subsegmental PA	0	0	0	0

MPA: main pulmonary artery; LPA: left pulmonary artery; RPA: right pulmonary artery.

## Discussion

The CT timing is critical for CTPA, especially for fast multidetector CT. It would be better to individually and precisely obtain the delay scan. In the present study, we optimized the protocol of CTPA by using the biphasic time-enhancement curves and the T_DELAY_ could be obtained by subtracting T_SD_ from T_CROSS_. Our data further showed that the personalized protocol could be used for CTPA examinations at 80 kVp with 10 ml contrast agent to obtain sufficient image quality and a low iodinate load (10 ml). PE in the segmental PA could be clearly shown using the optimized protocol.

The bolus tracking system and a test bolus are commonly used to start a scan^3,20,21^. However, the time obtained from those two techniques are not the scan delay^6^. The diagnostic delay is required to optimize the scan delay^6^. The diagnostic delay is related to several factors, including the injection duration, scan duration, and hemodynamics.^3^ In addition, the diagnostic delay may vary for different CT scanners.^3^ A equation was proposed by Bae^3^: T_DELAY_ = Time to peak contrast enhancement (T_PEAK_) − (1/2) × T_SD_. The T_PEAK_ for CTPA is determined by the injection duration (T_ID_) and contrast material arrival time (T_ARR_)^14^ and T_PEAK_ = T_ID_ + T_ARR_ −5^3^. He further demonstrated that this equation may work well in fast multidetector CT. For slow scans or scans with prolonged injection of the contrast agent, a modification is required because the T_PEAK_ and T_SD_ both increase. However, this equation is relatively complex. Various modifications have been reported in the literature.^22^ In our protocol, we found that T_DELAY_ is determined by T_CROSS_ and T_SD_. The T_DELAY_ calculated with our equation is simpler than that determined by using Bae’s equation. Moreover, our results showed that the image quality of the pulmonary arteries was better in test group than that in the control group (all score >3). The CT number of pulmonary veins in the upper and lower lobes in the test group were both significantly lower than those in the control group. Our optimized protocol could improve the image quality by diminishing the intervention of vein enhancement. Moreover, our data showed that PE in the segmental PA could be identified using this optimized protocol which indicated that this protocol was useful for PE detection.

CIN is another issue that limits the clinical application of CTPA, particular in patients with severe chronic kidney disease (CKD). Contrast volume reduction strategies have been used in CTPA^11,13,23–27^. Usually, 20–75 ml contrast agent were used in previous studies. To the best of our knowledge, few studies have used volume of contrast agent as low as 10 ml, in CTPA. The iodinate load in our study was lower than data previously reported^11,28^. The low tube voltage, such as 80 kVp can result in higher intravascular CT attenuation than 120 kVp^3,11^. Moreover, our scanning protocol may provide an accurate delay time, which is crucial for opacification of the pulmonary vasculature^11^. In addition, respiratory coordination and “ breath holding without taking the deep inspiration” were used during the scanning which can improve contrast enhancement in pulmonary arteries^29,30^. We used a small FOV, from diaphragm to 2 cm above the aortic arch, which may be one of the reasons for acceptable image quality using such a low iodinate load. Some strategies for iodinate reduction have also been recommended in literature^31,32^. Our optimized protocol using such a low volume of contrast agent is helpful for patients with CKD when they require CTPA examinations. However, for patients with BMIs > 28 kg/m^2^ or body weights > 80 kg, we speculated that a small additional volume of contrast media are required. Further evaluation are needed.

Iterative reconstruction at a low tube voltage, such as 80 kVp, has been widely used as a strategy for radiation reduction, especially for CTPA^2,26,33^. An 80 kVp was also used in our study. High pitch CTPA with 70 kVp in dual-energy CT or high-end CT also have been reported in several studies^28,34,35^. In our study, a 64 multidetector CT was used because it is more widely used in the world than dual-energy CT or high-end CT, which indicated that our optimized protocol could be widely used in CTPA examinations. Although the CT numbers in the test groups were lower than those in the control group, the image quality of pulmonary arteries were all sufficient for imaging diagnosis. Residual contrast material in the superior vena cava can result in streak artifacts^36^. Our results showed that the artifacts of SV and SVC were significantly decreased in the test group.

Our study also has several limitations. First, the sample size in the test group was relatively small, and our study was a single-center observational study. Second, patients with BMIs > 28 kg/m^2^ and body weights > 80 kg were not included in our study. Further studies are required to test whether our optimized protocol is also applicable in those patients. Third, our protocol may not be applicable for patients with severe asthma because it is difficult to measure the CT numbers of pulmonary veins. Consequently, it is difficult to obtain the time-enhancement curves in veins. Fourth, we did not compare our optimized protocol to the bolus tracking technique. Finally, our protocol may not be used for aortic or systemic arteries because there is no enhancement in those tissues.

In conclusion, we established an optimized protocol for CTPA examinations using biphasic time-enhancement curves. Our protocol could be used for CTPA at 80 kVp with 10 ml contrast agent to obtain improved image quality and a low iodinate load. Our optimized protocol may contribute to personalized CTPA examinations.

## Methods

### Patient selection

This prospective study was approved by the Institutional Review Board of the Affiliated Hospital of Nanjing University of Chinese Medicine (2018NL-085-01) and informed consent was obtained from each individual. The Declaration of Helsinki was followed during this study.

We enrolled 70 subjects who were suspected to suffer from PE. Subjects with body mass index (BMI) > 28 kg/m^2^, weight > 80 kg, III-IV cardiac function, severe arrhythmia, liver or renal failure or iodine allergy were excluded. They were randomly divided into two groups: the test group and control group. There were 25 men and 45 women. The mean age was 65.2 ± 13.4 years (age range, 27–88).

### CT protocol

All the patients in this study underwent contrast-enhanced CT with a 64-row GE Optima CT670 (GE Healthcare Tokyo Japan). The CT protocols are listed in Table 4. The patients were asked to perform “breath holding without taking the deep inspiration” during the scan to decrease the transient interruption of contrast arrival due to change in intra-thoracic pressures due to Valsalva maneuvers or changes in heart output. The test bolus in the control group received 10 ml nonionic iodinated contrast material (Omnipaque, 350 mg I/ml, GE Healthcare, USA) at a rate of 5.0 ml/s via a pump injector (Ulrich Missouri XD2001, Ulrich GmbH & Co.KG Germany) followed by 20 ml of normal saline. The monitoring scans started after the beginning of the injection and performed every two seconds. Then, we supervised the main pulmonary artery and obtained the time-enhancement curve. The T_DELAY_ was based on the curve and calculated as the sum of the contrast material arrival time plus an additional delay (also called diagnostic delay, 1–2 s). Then, the patients in the control group received 20 ml nonionic iodinated contrast material at a rate of 4.0 ml/s via a pump injector followed by 30 ml of normal saline.

**Table 4:** Scan protocol.

	Test group			Control group
Tube voltage	80 kVp			100 kVp
Tube current	100–400 mA			100–400 mA
Rotation time	0.5 s/r			0.5 s/r
Pitch	1.375:1			1.375:1
Noise index	11			11
BMI or body Weight (BW)	BMI < 24 and BW < 60		24 ≤ BMI < 28 or 60 ≤ BW < 80	BMI < 28 and BW < 80
Contrast agent	Iodixanol (270 mgI/ml)		Omnipaque (350 mgI/ml)	Omnipaque (350 mgI/ml)
Injection ratio	5 ml/s			4 ml/s
Pre-scan	5 ml contrast agent + 45 ml normal saline			10 ml Omnipaque + 20 ml normal saline
Delay scan methods	Biphasic time-enhancement curves			time-enhancement curve of artery
Enhanced scan	10 ml contrast agent + 40 ml normal saline			20 ml Omnipaque + 30 ml normal saline

BMI: body mass index; BW: body weight.

**Figure 6:**
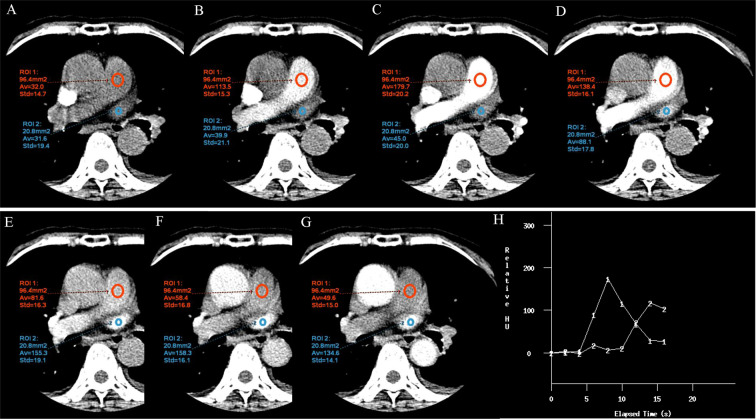
The biphasic time-enhancement curves. The region of interest was placed on main pulmonary artery and vein (**A–H**). The time-enhancement curves were obtained automatically (**H**). Line 1 was the time-enhancement curves of pulmonary artery; Line 2 was the time-enhancement curves of pulmonary vein.

The CT protocols in the test group are listed in Table 4. The test group first received 5 ml nonionic iodinated contrast material (Omnipaque, GE Healthcare, USA or Iodixanol, Yangtze River Phar Co., Ltd, Taizhou, China) via a pump injector followed by 45 ml of normal saline. The regions of interests were placed in both the pulmonary artery and vein. Then, the biphasic time-enhancement curve was obtained by measuring the enhancement within a region of interest (Fig. 6). The monitoring scans were stopped when the ascending aorta was enhanced. The two curves had a meeting point and the time at this point was set as T_CROSS_. The scan delay for the test group was calculated based on the biphasic time-enhancement curves. The scan should be finished before the intersection. In addition, we also calculated the T_SD_. BMI and weight both affect contrast enhancement^8^. Subjects with BMIs <24 kg/m^2^ and weights <60 kg received 10 ml iodixanol (270 mg I/ml). Subjects with BMIs between 24 and 28 kg/m^2^ and weights between 60 and 80 kg received 10 ml Omnipaque (350 mg I/ml).

The CT scanning parameters were as follows: tube voltage, 100 (control group) or 80 (test group) KVp; tube current, 100–400 mA; helical pitch, 1.375 ; section thickness, 0.625 mm; gantry rotation time, 0.5 s. The adaptive statistical iterative reconstruction (ASiR) was 50%, which mean 50% filtered back projection (FBP) plus 50% ASiR.

### Image quality evaluation

Two chest radiologists (HZ and BQ) with 5 and 10 years of CT experience, respectively, independently evaluated the subjective image quality. All images were evaluated in the workstation. We obtained the maximum intensity projection (MIP) and curved plane reformation (CPR) images. The following vascular enhancement was evaluated quantitatively: MPA, LPA and RPA, left upper and lower lobe pulmonary artery, the apical segment in the left upper lobe and the companion vein, the basal segment in the left lower lobe and the companion vein. The regions of interest (ROI) measured with a size of 80 ± 5 mm^2^ in the MPA and 60 ± 5 mm^2^ in the LPA and RPA. If there was a lesion, measurements were performed in adjacent slices or the contralateral vessel. The CT numbers of the bilateral pulmonary lobes and paraspinal muscle were also measured with an ROI of 30 ± 2 mm^2^ at the level of the pulmonary trunk. We calculated the SNR using the following formulas: SNR = CT number of target artery/background noise, CNR = (CT number of target artery - CT number of paraspinal muscle)/background noise. Three independent measurements were performed, and the mean was calculated.

We also subjectively evaluated the image quality. The overall image quality of the PA was scored on a five-point scale: 5, good to excellent, the arterial branches are clearly demonstrated and the pulmonary veins were not enhanced; 4, good, pulmonary artery was clear and the pulmonary veins were indistinctly observed; 3, the enhancement of arteries and veins were similar; 2, the enhancement of veins were higher than the arteries; 1, nondiagnostic quality, the images could not be used for diagnosis because of poor contrast opacification. The artifacts of SV and SVC were also recorded (Yes or No). The quality of the lung window images was scored on a three-point scale: 1, excellent image quality; 2, good image quality; 3, a nondiagnostic examination.

### PE detection

Two chest radiologists (with 8 and 12 years of CTA experience, respectively) who were blinded to the patient medical histories and clinical data independently evaluated the images. PEs were considered if there were luminal filling defects or non-visualization of segmental pulmonary and subsegmental arteries compared to the contralateral side. We counted the number of PE in the MPA, LPA, RPA, lobar PA, segmental PA and subsegmental PA.

### Statistical analysis

The SPSS for Windows (version 19.0; SPSS Inc., Chicago, IL, USA) was used in statistical analysis. We first performed Kolmogorov-Smirnov test for normal distribution of the data. For those data with normal distribution, the independent t-test was used for comparing continuous variables. Mann-Whitney U test was used for continuous data with abnormal distribution. The chi-squared test or Fisher’ test was used for the categorical variables. The significance level was set at P < 0.05.

## Supplementary information

Supplementary information.

## Data Availability

All data generated or analyzed during this study are included in this published article (and its Supplementary Information Files).
