# Supplementary material for: An optimized test bolus for computed tomography pulmonary angiography and its application at 80 kV with 10 ml contrast agent
Source: Sci Rep. 2020 Jun 23;10:10208. doi: 10.1038/s41598-020-67145-9 (PMC7311447; doi:10.1038/s41598-020-67145-9)
Supplement: Supplementary file 1 — Supplementary information. [file 41598_2020_67145_MOESM1_ESM.pdf]

An optimized test bolus for computed tomography pulmonary angiography and its application at 80 kV with 10 ml contrast agent

Huiming Wu, MS,[1] Xiao Chen, PhD, [1] Hao Zhou, MS,[1] Bin Qin, MS, [1] Jian Cao, MS,[1] Zhaochun Pan, MS,[1] Zhongqiu Wang, MD, PhD, [a]

1. Department of Radiology, the Affiliated Hospital of Nanjing University of Chinese Medicine

Huiming Wu and Xiao Chen contributed equally to this work.

Corresponding author: Zhongqiu Wang, Department of Radiology, Affiliated Hospital of Nanjing University of Chinese Medicine, 150 Hanzhong road, Nanjing 210029, China, Phone: 086-025-86617141, Email: zhqwang001@126.com

Supplementary information

Table s1 Patient Characteristics

	Test group (n=35)	Control group (n=35)	P values
Gender (Male/Female)	12/23	13/22	0.803
Age	63.3±15.5	66.5±11.4	0.380
Height	161.6±8.3	163.5±7.6	0.325
Weight	57.9±10.7	57.6±13.3	0.923
BMI	22.1±3.6	21.4±4.2	0.446

Table s2 Delay scan obtained from our protocol and test-bolus method

		Scan duration (s)	Delay scan(s)	Test-bolus
Control	Patient A	3.41	11	
Test group	Patient B	2.77	7	6
	Patient C	2.63	4.5	5
	Patient D	3.17	8	9
	Patient E	3.18	5.5	7
	Patient F	2.27	3	5
